# Fading vision: knowledge translation in the implementation of a public health policy intervention

**DOI:** 10.1186/1748-5908-8-59

**Published:** 2013-06-04

**Authors:** Laura Tomm-Bonde, Rita S Schreiber, Diane E Allan, Marjorie MacDonald, Bernie Pauly, Trevor Hancock

**Affiliations:** 1 University of Victoria, School of Nursing, P.O. Box 1700 STN CSC, Victoria, British Columbia, Canada; 2 University of Victoria, School of Public Health and Social Policy, P.O. Box 1700 STN CSC, Victoria, British Columbia, Canada

**Keywords:** Core public health functions, Public health reform, Implementation, Knowledge translation

## Abstract

**Background:**

In response to several high profile public health crises, public health renewal is underway in Canada. In the province of British Columbia, the Ministry of Health initiated a collaborative evidence-informed process involving a steering committee of representatives from the six health authorities. A Core Functions (CF) Framework was developed, identifying 21 core public health programs. For each core program, an evidence review was conducted and a model core program paper developed. These documents were distributed to health authorities to guide development of their own renewed public health services. The CF implementation was conceptualized as an embedded knowledge translation process. A CF coordinator in each health authority was to facilitate a gap analysis and development of a performance improvement plan for each core program, and post these publically on the health authority website.

**Methods:**

Interviews (n = 19) and focus groups (n = 8) were conducted with a total of 56 managers and front line staff from five health authorities working in the Healthy Living and Sexually Transmitted Infection Prevention core programs. All interviews and focus groups were digitally recorded, transcribed and verified by the project coordinator. Five members of the research team used NVivo 9 to manage data and conducted a thematic analysis.

**Results:**

Four main themes emerged concerning implementation of the CF Framework generally, and the two programs specifically. The themes were: ‘you’ve told me what, now tell me how’; ‘the double bind’; ‘but we already do that’; and the ‘selling game*.’* Findings demonstrate the original vision of the CF process was lost in the implementation process and many participants were unaware of the CF framework or process.

**Conclusions:**

Results are discussed with respect to a well-known framework on the adoption, assimilation, and implementation of innovations in health services organizations. Despite attempts of the Ministry of Health and the Steering Committee to develop and implement a collaborative, evidence-informed policy intervention, there were several barriers to the realization of the vision for core public health functions implementation, at least in the early stages. In neglecting the implementation process, it seems unlikely that the expected benefits of the public health renewal process will be realized.

## Background

There is longstanding unease in Canada about the adequacy of public health infrastructure to address major public health concerns [1,2]. In the early 21st century, several public health emergencies highlighted the need for public health reform. In particular, water contamination events [3] and the Severe Acute Respiratory Syndrome (SARS) outbreak in Toronto [4] drove home the need for public health renewal [5]. The centerpiece of the province of British Columbia’s (BC) approach to reform is a Framework for Core Functions in Public Health (CF Framework) [6]. It identifies the basic public health services and supports that Health Authorities (HA) are expected to provide to their communities (see Additional file 1).

The launch of this Framework provided the impetus for developing a research program, Core Public Health Functions Research Initiative (CPHFRI) [7], to study the implementation and impact of public health system renewal in BC and beyond. CPHFRI includes the Renewal of Public Health Systems (RePHS) project in which we study the implementation of the CF Framework in BC and the Public Health Standards in Ontario (ON), through two exemplar core public health programs: sexually transmitted infection prevention (STIP) and chronic disease prevention, known in BC as healthy living (HL). The findings reported in this paper are derived from RePHS BC phase one data on the introduction and early implementation of the CF Framework and the two core programs from the perspectives of public health managers and practitioners.

### Implementation and knowledge translation

Implementation has been generally studied as a component of a larger change process, such as the diffusion of innovations [8,9]. Implementation refers to a process of translating intentions into action with many possible outcomes, including degrees of both successful and unsuccessful implementation [10,11]. These intentions are reflected in policies, plans, technologies, programs, and innovations. In public health, like other areas of healthcare, there has been movement toward evidence-based practice, programs, and policies [12], and several terms have emerged to describe the process by which evidence is translated into practice, such as knowledge translation (KT), knowledge transfer, and knowledge exchange [13].

The KT literature, which has grown considerably since 2000, overlaps with older implementation literature. Both KT and implementation theory are about putting knowledge into practice, but KT is explicitly focused on research evidence and the relationship between researchers and knowledge users. The older implementation literature focuses more on implementing innovations, policies, and programs, and influences on that process. Both bodies of literature inform our analysis.

From their meta-narrative systematic review of the diverse literature across 13 research traditions on diffusion of innovations in healthcare organizations, Greenhalgh *et al.*[8] developed a comprehensive conceptual framework of factors influencing dissemination, adoption, implementation, and sustainability. The main categories of influence on these include: the nature of the innovation; system antecedents for innovation; communication and influence; the outer context; the inner context; system readiness for innovation; characteristics of the adopters; and the implementation process. In this framework, implementation is one stage in the entire diffusion process, but factors influencing each stage are not distinct and influence all stages.

There is widespread acknowledgement of the importance of evidence-based practice and policy, yet the research-practice gap remains a significant problem [14,15]. Theories, frameworks, and models have been developed to guide and explain the implementation and KT process [8,15-18]. KT is a process of closing the gap between the evidence generated in research and its application for frontline health systems delivery [19]. Here, ‘application’ or what we call ‘implementation’ is considered part of the KT process. Unfortunately, implementation and KT frameworks have not been used widely by organizations prospectively to plan implementation of new policies or programs [16,18]. We know that few policies and programs in the ‘real world’ are fully implemented as intended [20], but we do not know how the use of KT or implementation frameworks might improve the process.

In recognition of this challenge, the BC Ministry of Health developed a Core Functions Implementation Process [21] to guide the implementation of the Core Functions Framework [6] in BC, which can be understood as an embedded KT process. In other words, KT was integrated into the policy intervention itself as illustrated below.

### The core functions implementation process—embedded knowledge translation

A provincial steering committee was created to develop, oversee, and guide the CF framework implementation. This committee, composed of Ministry and HA representatives, commissioned and approved evidence reviews for each of the core programs. Provincial teams, which included HA representatives, used the evidence reviews to develop 21 model core program papers (MCPP). At this point, responsibility for implementation was turned over to HAs. Leaders within each HA were identified for each core program and they convened working groups to conduct gap analyses in which existing services were examined in relation to the evidence-informed practices described in the MCPPs. The working groups were to develop performance improvement plans (PIPs) to implement the MCPP strategies that addressed identified gaps in service. If the HA was already implementing some of the best practices described, they could continue to offer those services if other priorities were not identified and implement new services to address gaps.

Expectations for Framework implementation were communicated to the HAs via a General Letter of Expectation (GLE) from the Ministry. Given that public health services represent only a small part of services provided by HAs, they were not the major focus of the GLE. Once HAs conducted their gap analyses and developed PIPs, these were to be made publically available on each HA’s website.

### Purpose of study

The implementation of this major policy initiative was expected to have a significant impact on public health practice in the province, in part through its embedded KT process.

Few implementation studies have focussed explicitly on developing a qualitative understanding of the experiences of those who are actually involved at the front lines. In this paper, we focus on presenting the perspectives of those responsible for implementation on the ground. Specifically, we explore the collective experiences of HA managers and front line staff with the early implementation of the CF Framework within the STIP and HL exemplar programs in BC. An analysis of the differences between HAs and specific subgroups of participants is beyond the scope of this paper and will be presented in subsequent publications. First, we present a thematic analysis, and then discuss findings in relation to key influences on implementation drawing on Greenhalgh *et al.*’s framework on the diffusion of innovations in health service organizations [8]. Knowledge about the implementation experience and the challenges encountered by practitioners and managers may help inform improvements in both the policy intervention and the implementation process.

## Methods

### Sampling

The data used for this paper were obtained during phase one of the larger RePHS project (see Additional file 1 for a description of the overall research project and its methodology). We conducted interviews and focus groups with BC public health practitioners and managers involved in STIP and HL about their early implementation experiences. Consistent with most qualitative research methodologies, we used purposive sampling [22]. A key representative within each HA extended an invitation to participate to managers and front line staff who had experience with planning or delivery of STIP or HL. If interested, representatives contacted the research team directly.

### Data collection

Questions were based on sensitizing concepts from Greenhalgh *et al.*’s framework as described previously [8] and reviewed for relevance and wording with knowledge user partners. Topics included: knowledge about the CF Framework and STIP/HL implementation, how evidence informed practice, integration of an equity lens into STIP/HL activities, public health governance, leadership, partnerships, and contextual influences on each of these. Interviews lasted approximately 90 minutes. Similar interview guides were used for both interviews and focus groups.

For pragmatic reasons (*e.g.*, HA size and staff workload), as suggested by knowledge user partners, we conducted focus groups with front line staff, and individual interviews with managers. From November 2010 to June 2011, individual interviews (n = 19) and focus groups (n = 8) were conducted with a total of 56 HL and STIP managers and front line staff from four of five regional HAs and the provincial HA. Data collection was conducted in-person (five interviews, six focus groups) or by teleconference (14 interviews, two focus groups) based on HA preference. Ethics approval for the larger project was received from the University of Victoria and each participating HA. A review of interview and focus group data suggest that the data were similar in depth and scope, although individual focus group responses were shorter than interview responses. However, focus group participants built on other participants’ responses, resulting in a collective response to each question similar in detail to those of individual interviewees.

### Analysis

Interviews and focus groups were digitally recorded, transcribed verbatim and verified. We conducted a thematic analysis using NVivo 9.0 to manage the data. The team listened repeatedly to audiotapes, and read transcriptions of the interviews and focus groups [23]. We inductively coded the data using the constant comparative method [22,24] beginning with line-by-line coding, ultimately aggregating codes at higher levels of abstraction. Analysts wrote memos throughout, recording insights and making notes about relationships among the emerging categories, later verifying these in the data. Several coders analyzed the same interviews and developed an initial coding framework to be used by the whole team for coding subsequent interviews. The coding framework evolved as analysis progressed, facilitated by regular team meetings to discuss and agree on emerging themes and resolve discrepancies in coding.

Knowledge users on the research team provided feedback throughout the process which helps to ensure that findings are accurate and credible [25]. The themes described below have been presented to and discussed with HA team members and research participants in several venues.

## Results

Four main themes emerged regarding participant experiences with implementation of the CF Framework and the two core programs. The themes were: ‘you’ve told me what, now tell me how’; ‘the double bind’; ‘but we already do that’; and ‘the selling game*.’* We discuss each of the themes with illustrative quotations. We applied gender-neutral pseudonyms to quotations to protect confidentiality and edited quotations slightly to enhance readability and meaning (*e.g.*, removal of instances of um and repetitive phrases).

### You’ve told me what, now tell me how

The CF Framework was designed to be a guide, not legislated. The public health renewal process was collaborative involving representatives from government, HAs, and other stakeholders. The government acted in a stewardship role, responsible for overseeing but not directing the process. Because the HAs in BC operate as independent organizations, the vision was that each would implement programs according to their unique governance structures, population needs, and resources. HAs were asked to conduct gap analyses and develop PIPs for each of the core programs, using the evidence reviews and MCPPs. Shifting responsibility for implementation from the provincial to the HA level meant involving more, and different, individuals in the CF process. Some people assisting at this point were not involved in earlier stages of the framework process, yet were now being asked to assess the current state of services and develop new plans. Many claimed not to have a clear understanding of the vision, intent, or meaning of the Framework or the implementation process. Their focus was on completing the gap analysis and PIP without really knowing how these activities fit into the larger vision of the framework. They felt as though they were going through the motions of implementation without understanding why, as reported by this manager:

‘I don’t know, maybe because in the past I’ve been involved in doing their [the Ministry’s] research and pulling this together and being very specific about why we’re doing something. Whereas this way, the papers are already written and you are just sort of relying on what you get. I know this sounds really odd. But if someone else is doing it for you and then you’re rolling it out.’ (Chris, Manager)

This participant is suggesting that there is something inappropriate about practitioners who are not involved in the development of the plan being responsible for rolling out someone else’s plan. In such a situation, it is possible that the commitment needed to implement as intended is missing. Without an understanding of the larger vision, more questions than answers arose in determining how to operationalize programs. For example, participants raised questions such as: ‘Is my staff responsible for this? What are common indicators across the region and province and shouldn’t we be working towards these? How are other HAs doing this? Some of the activities that we do, cross into other core programs, so how do we deal with that? If we can’t do everything in the MCPP, where should we focus our attention?’ They perceived a lack of guidance in answering their questions. As one participant put it:

‘And it just seemed crazy to me that the provincial government was unrolling something without consultation with them [front line staff]. And so they [the staff] have to wait to see what they are doing but how, if they haven’t consulted with them. I mean they’ve never consulted with us to see what they do for obesity prevention in schools and, you know, you just think—who’s actually on the ground?’ (Charlie, Front-line staff)

Clearly, some staff felt left out of the process despite their belief that they had important contributions to make. Although participants thought a few front line staff may have been involved in the process, there was concern that this was inadequate because of the variety of roles and the inability of one person to reflect accurately the diversity of front line perspectives.

Some participants reported that many people in their organization had never heard of the CF Framework. For example, one participant said, *.’*…what was interesting, you know, talking about model core programs and people didn’t know what I was talking about’ (Cameron, Manager). It appeared that the philosophical vision and intents were not adequately communicated throughout the organization, nor were these staff members engaged in a process to develop a shared vision. Thus, the ‘how’ of implementation became somewhat irrelevant in relation to the vision of the Framework. Instead, they focussed on what they thought they needed to do rather than understanding how or why.

### The double bind

Those working in HAs were asked to implement 21 core programs with few, if any, additional resources to support the endeavour. If additional resources were available, this was not evident to all managers and front line staff. Initially, people felt overwhelmed by the enormity and complexity of the Framework’s implementation and struggled to find strategies to make it more manageable. Most HAs responded by clustering core programs into related groups. For example, in one HA, infant and child health was bundled with reproductive health, while HL and healthy communities were clustered with mental health promotion and preventing the harms of substance use. Others clustered programs into age-related groupings. The intent was to reduce the number of programs to a manageable number or to consolidate services provided to specific population groups.

In struggling to comprehend the scale of the public health renewal project, some participants searched for supports and resources to help them. Many stated that public health budgets (and departments) were cut, resulting in job losses, non-replacement of vacant positions, and shrinking programs, rather than the hoped-for expansion and renewal of public health. As one manager noted, it was a challenge implementing core programs in light of organizational cost cutting measures at the same time that other public health crises were emerging:

‘But then all these things in the past year and a half have happened that make that process [*i.e.*, implementation] so much harder and make things take so much longer. So instead of, ‘it’s no problem to get a gap analysis and performance improvement plan done in a year*,’* now things can take quite a bit longer. There is just kind of one impact after another. So right after I started there was fiscal restraint because of the economic times we were in, so we had quite a lot of budget cuts so people lost their jobs and we couldn’t travel. So that hampers getting teams together when you can’t travel and then people are losing their positions that were maybe a lead for a core program or who were a significant personnel on that team and that changes the make-up of your team and the ability to get it done. And then H1N1 happened....’ (Casey, Manager)

Participants perceived that with budget cuts there was little hope of HAs putting into place an effective implementation strategy and that this magnified the inadequate surge capacity of the system to deal with public health emergencies, such as the H1N1 epidemic. In the face of such crises, other public health programs got put on hold.

Without access to financial and other resources, many did not find administrative support for the CF process, seeing a mismatch between the stated intentions of the Ministry and the means for implementing public health renewal. Being responsible for changes without having the means to enact them put managers in a double bind and left people feeling frustrated and burdened. Many felt that public health, in particular, was being unfairly targeted for budget cuts, as reflected below:

‘But I think for me, one of the issues has been the pressure on the limited resources and I’m still not clear why they always pick on the little guy right? And that seems to be, whether people believe that the money is more available [in public health], I don’t know why you’d pick on the area that has the least amount of dollars. I can remember when one of my directors, I said to him, ‘Instead of just taking little bits from me, why don’t you just take the whole program because you’re talking about, you know, if we have deficits of a program, this may be 5 or 6 million. Well if you have to cut that much just wipe it out ‘cause that’s what you’re doing on a gradual basis. You’re letting us kind of haemorrhage. You may as well take it away.” (Jamie, Manager)

Even in places where implementation began well, and the gap analyses and PIPs were completed in a timely fashion, not having adequate resources to see it through hampered the process and demoralized practitioners.

Some sought direction regarding which, if any, of the 21 programs were a priority within the HA, but did not find much guidance. Many respondents suggested that they were unable to determine priorities because these were shifting within government. At the time the CF framework was introduced and people in HAs were awaiting a government announcement on the HL core program, people talked about the negative impacts of the H1N1 pandemic, a provincial election, an economic downturn, and restructuring within HAs. The ‘change fatigue’ they reported was a significant barrier to implementation. Although work on core functions continued in some HAs, in other cases the implementation of some core programs was put on hold while waiting for guidance about priorities and the necessary resources to carry through with it.

As a consequence, staff in some HAs made their own priority assessments. In the perceived absence of Ministry and HA leadership and funding in support of core functions, they believed that full implementation of the vision of the CF Framework was severely hampered, as implied by this front-line staff member:

‘Just in terms of the core functions, we would hope that there’s some bite to the actual functions. Like, just putting up policy is not helpful. There needs to be dollars attached to the policy and there needs to be buy in from management. Then it’s helpful to us. Then they feel the impetus to give us the information and knowledge translation is improved.’ (Reese, Front-line staff)

### But we already do that

Although the vision of the framework was to define the core public health functions that HAs should be addressing, as well as to provide relevant evidence and guidelines for what should be included within each core program, to many participants, the framework meant starting from scratch and implementing a new program. The Ministry and steering committee never envisioned that all current programs would cease and new ones begin and yet, that message never filtered down to many of those engaged in gap analyses or program implementation. In talking about this problem, on participant said:

‘The information, it hasn’t been very clear and it hasn’t been big picture, and I think a lot of people have been, need the big picture to get the small picture’ (Quinn, Front-line staff).

Some staff questioned the intent of core functions because to them CF implementation meant that the good work they were already doing was not being acknowledged. For example, one participant noted,*.*‘I would argue that we were already implementing best practices’ (Eden, Manager). Although some participants did recognize that the intention was about making sure the gaps were covered and, if necessary, services were enhanced, they did not believe that others understood this, as reflected below:

‘I think what was missing was a strong awareness of how core functions is a part of everything that we do in the HAs and how the core functions evidence can be used to support planning and decision-making and how it doesn’t have to be a project that’s separate from all of the other work. It’s actually integrated with all the other work and supporting the other work and vice versa. So that to me was kind of the big thing.’ (Sidney, Manager)

Many participants did not recognize the connection between their current programming and the intentions of the CF Framework. Repeatedly, we heard participants say that they were already providing the services outlined in the MCPPs without understanding that the intent was never to start from scratch. For example, in reference to STIP, one manager noted:

‘And you know, while sitting here and thinking, like, why haven’t we focused on this with our core function work that we have done, I think it is because it is a good program already in place. So I think that is probably one of the reasons why STIP probably hasn’t made it into any of the implementation plans of core function groups.’ (Morgan, Manager)

The theme ‘But we already do that’ suggests that many participants believed that best practices were already in place and thus could not understand why a new framework or core program was needed. Clearly, the intent of the CF Framework was neither understood by, nor explicitly communicated to, all staff and this led to lack of commitment to the entire process by some staff.

### The selling game

A major challenge to implementation noted by participants was competing HA priorities. When decisions had to be made, some participants said that everything associated with the CF Framework fell to the bottom of the list. This was because they perceived no consequences from the Ministry for non-implementation and that ongoing resources beyond the initial funding were not provided either by the Ministry or the HAs. The political will within the HA to implement the Framework was not always there, as this participant explained:

‘So they [HA employees], so they like that [the vision of the CF Framework] in principle. [They] like, the concept of it, being able to show that we’re all in this together. But I think that people recognize that change is political, and the political will wasn’t there, you know, in the organization. So there was always scepticism as to whether it would be implemented.’ (Pat, Manager).

The process thus became a marketing exercise for those responsible for implementation in which they ‘polished the rhetoric’ to produce buy-in from others to work on the program, be committed to the process, and move forward despite all of the odds stacked against them. This participant explains her experience:

‘It’s already kind of a selling game. You have to [tell them], you know, why is this important? Why are we doing this? And yes we are mandated to do the core programs, but why is this actually going to help your work and improve our services to our clients? You kind of have to sell that because everyone is so busy and people are not going to give up another part of their job to do this work. It is an added on part of their work right, so it’s making that sell and then we can work on this for six months and not sure where it will go, right?’ (Leslie, Manager)

The purpose of the selling game was to generate support in HA areas outside of public health for a policy that did not appear to carry the same political weight as other government or HA initiatives. Participants saw no guarantee that work done now would still be relevant in six months. Thus, getting buy-in in an ever-changing environment was a challenge. For some, it was a matter of selling the work associated with the program. To make it work, however, participants believed that there needed to be strong leadership at all levels in the organization, not only in supporting CF implementation, but also in advocating for the role of public health within the HA. One participant summed this up well:

‘I think that the leadership style needs to be collaborative and inclusive and then what I mean by that is reaching out to other programs outside of public health and making the links for them. Like why core programs is important. How it can help support them in the work they are already doing. Why it benefits the populations we serve to be integrating this evidence and these practices into our work. So, it needs to be someone who understands the importance of that and who is willing to make time for that. Cause it’s time-consuming. It’s about conversations really, and relationships.’ (Jessie, Manager)

Communicating a vision is an important leadership tool for implementation. In some instances, the managers’ sales pitch to front line staff may not have included the vision, but instead focused on the gap analysis and the PIP. Without understanding the vision, it was difficult for mid-level managers and front-line staff to understand or buy into the significance of these activities. In other instances, however, there was no sales pitch, so people were left to implement activities without understanding why.

In summary, four themes emerged from the analysis that explained participants’ experiences with implementation of the core programs. Although some participants did report positive aspects of the CF process and framework, overall, the introduction and early implementation of the CF Framework was not wholeheartedly embraced by many participants and resulted in what appeared to be a rocky start to the CF process.

## Discussion

The CF Framework was a comprehensive, evidence-informed, collaborative initiative intended as an embedded KT process to renew public health in BC. Many participants saw value in this framework and acknowledged the importance to public health. Nonetheless, we found that many factors known to impede successful implementation were present at the time the CF Framework was introduced in BC. Without addressing these challenges, the vision for public health renewal in this province may not be realized.

One limitation of our analysis is that it draws only on a subset of data being gathered to study the implementation of this policy intervention. We recognize, however, that implementation is often a long and protracted process and that what influences the adoption and implementation of innovations may change at different points in the process [8]. Additional data gathered during phase two (interviews with senior executives in the HAs) and phase three (follow-up interviews with front line staff and managers) may reveal that influences may change as the implementation process evolves over time.

Greenhalgh *et al.* argue that ‘the success of implementation (and the chances of sustainability) are critically dependent on the attributes of the innovation, the behaviour of individual adopters, the nature of communication and influence, and various structural and sociological features of the organization and its wider environment’ [8, p. 176]. In our discussion, we focus on our empirically derived findings in relation to these elements of their framework.

### Intervention characteristics

The important characteristics of the intervention that appeared to influence early implementation include the intervention source, relative advantage, complexity, and reinvention. Perceptions that an intervention is developed externally, or is seen as centrally driven, can negatively affect implementation success, particularly if there is not a transparent decision-making process or there is limited input from users [16,26]. Despite HA involvement in developing the CF framework, some participants were not aware of this and did not perceive that there had been sufficient consultation with those responsible for implementation. This appeared to result in a low level of commitment to development and implementation of new practices from the MCPPs. Some perceived the Framework as a directive from above, as reflected in the theme ‘you’ve told me what, now tell me how.’

There must be a relative advantage of an innovation to all stakeholders for successful implementation [8,9]. Many participants did not see a clear benefit of the CF framework or MCPPs over current practice. In fact, they believed the MCPPs reflected what they were already doing (but we already do that!). The seeming similarity of the MCPPs to what was already going on led some participants to dismiss the MCPPs as redundant. Relative advantage alone, however, is not sufficient for implementation. Innovations often go through a long period of negotiation, meaning-making, contestation and reframing, all of which may influence the perceived advantage of an innovation and thus its adoption and implementation [8]. Because participants were at the beginning of this protracted process, there are still opportunities for improving implementation over time.

The perceived complexity of an intervention will negatively influence implementation [8]. If the innovation can be reinvented (*i.e.*, adapted or modified) then implementation is more likely to occur [9]. In ‘the double bind’ many participants felt overwhelmed by the complexity of the CF Framework and managed this by clustering programs to reduce the demands of implementation. Whether this adaptation will improve the success of implementation over the longer term remains to be seen.

### Outer context

Factors in the outer context influencing implementation included: incentives and mandates at the provincial level, and environmental stability. As described in ‘the selling game*,’* participants identified few incentives in support of implementation. There was no strong mandate or political directive to ensure implementation and evidence suggests that such mandates are most effective when accompanied by a dedicated funding stream [27]. Overall, there was limited accountability for action, no perceived consequences for lack of implementation, and no funding to support implementation. The requirement to post the PIP publically was not tracked by the Ministry and not enforced. Although a requirement for public reporting can motivate compliance with directives in an organization [28], not if there are adversarial relations between the reporting entity and the organization to which it is responsible [10]. Some participants perceived the relationship between the Ministry and their HA as adversarial.

Environmental stability or uncertainty can influence the implementation process, although the evidence is limited [29,30]. Changes in the external environment have a much smaller influence than structural characteristics of the organization [29]. Nonetheless, participants talked about instability in the external environment created by significant organizational restructuring in some HAs, shifting policies and priorities of the provincial government, a provincial election that put CF implementation on hold, and the H1N1 pandemic that diverted both the resources and the practice of public health away from CF implementation.

### Inner context

In the inner or organizational context, influences on implementation include: system antecedents for innovation, *i.e.*, structural characteristics of the organization and a receptive context for change; and system readiness for innovation, including tension for change and innovation-system fit. Structural characteristics relate to such things as functional differentiation or internal division of labor, and the centralization versus dispersion of decision making [8,26]. Devolved decision making in the organization to teams on the ground contributes to successful implementation [30]. In this study, although there were working groups who conducted gap analyses and developed PIPs, they did not necessarily have decision making authority around program implementation. Front line staff, in particular, did not believe they had decision making authority in this regard.

Prior to regionalization in BC in the 1990s, public health units operated independently of the healthcare system at large with strong public health leadership [31]. Public health budgets could never be raided by the acute care system. Currently, public health is subsumed by the regional HAs, each of which structures public health somewhat differently [31]. That structure influences the power and authority of public health leaders, as well as the visibility and priority given to public health functions. For example, in some HAs, there is no public health representation at the senior executive table to bring a public health voice to decision making. Medical health officers in some HAs have no line authority or control of budgets, nor do they make decisions about core program implementation and the deployment of public health staff. Participants perceived that, without this, public health was marginalized and often invisible within the system, and that public health was unfairly targeted for cost cutting measures. This perception was evident in the themes ‘you’ve told me what, now tell me how’ and ‘the selling game.’

If an organization has a receptive context for change, then it is more likely to embrace new ideas and support the implementation of innovations [8,32]. Key components of this context include strong leadership and a clear and strategic vision [32,33]. Many participants perceived a lack of strong public health leadership within the organization. The lack of resources to implement core functions adequately was seen by participants as a concrete reflection of the lack of leadership to advocate for the role of public health, and consequently the low priority of public health in the system. Many participants did not perceive that there was a strategic vision for CF implementation in their HAs. If senior executives did have a strategic vision, it was not well communicated to front line staff and managers.

When practitioners believe they are already engaged in best practices (but we’re already doing that!), leadership may be important to push staff ‘to break out of the convergent thinking and routines that are the norm in large organizations’ [8, p. 13]. It may encourage staff to reflect critically on whether they are truly implementing best practices, or just engaged in business as usual.

Elements of system readiness for innovation included tension for change, innovation-system fit, and relative priority. Tension for change is the extent to which those involved perceive the current situation as intolerable or requiring change [34]. This can be fostered with effective communication strategies that create dissatisfaction with current practice, cultivate commitment, and reduce resistance. A tension for change was not reflected in most participants’ experiences and we found no efforts to cultivate tension. In fact, many believed that they were already engaged in practices outlined in the MCPPs (but we already do that!). Furthermore, participants were already experiencing change fatigue with many new initiatives and rapidly shifting priorities, as reflected in the theme ‘you’ve told me what, now tell me how.’ Commitment to implementation was hard to muster when people believed that six months down the road another initiative would be announced, requiring yet another change in their work.

### Innovation-system fit

There is strong evidence that when there is a good fit between the innovation and organizational values and norms, people are more likely to embrace the innovation [9,10,34]. Participants believed that they were already engaged in best practices outlined in the MCPPs, and they saw value and strength in the CF framework. This suggests that there was already a good innovation-system fit. Clearly, this was not sufficient for participants to embrace wholeheartedly the CF framework. A shared perception of the importance of the intervention’s implementation within the organization and its relative priority predicts implementation effectiveness. When staff members perceive that implementation is a key organizational priority, and it is promoted, supported, and rewarded, then a climate in support of implementation is strong [10,35]. In ‘the selling game’, participants made it clear that they did not perceive core program implementation as a priority in the organization, relative to competing priorities.

### Communication and influence

Intra-organizational networks and communications are important influences on successful implementation. Social networks support knowledge circulation in an organization. Support for teamwork is therefore important to facilitate the development of shared meaning, values, and vision in regard to the innovation [8]. This requires stable teams over time. In the theme ‘you’ve told me what, now tell me how,’ it is evident that teams were not stable and that staff changes greatly affected how people understood the intervention and their role within it.

It is possible that effective communication strategies might have mitigated these effects. We know that communication across the organization is essential for effective implementation [36]. In general, staff members need to be well-informed, and to understand the mission and goals of an intervention if implementation is to be successful [8]. It is evident that many participants did not have an understanding of the vision, intent, or meaning of the CF framework or the implementation process. A more consistent and organization-wide communication strategy might have better set the stage for subsequent implementation, perhaps by helping to create tension for change. There is some evidence to support a narrative approach to communication across the organization [37] in which a ‘purposive construction of a shared and emergent organizational story of ‘what we are doing with this innovation’ can serve as a powerful cue to action’ [8, p. 15].

### Implementation process

Greenhalgh *et al.*’s framework identifies the following leadership and management factors as important in enhancing implementation: top management support and advocacy of the implementation process, alignment of the innovation with the goals of top and middle management, and active engagement of leaders in the process. As reflected in ‘you’ve told me what now tell me how’, ‘the double bind’, ‘and the selling game’, it was clear that many participants did not perceive top management support of, advocacy for, or engagement in the implementation process. Participants suggested that the goals of upper level management in the HAs do not align with the goals of public health or the CF Framework. Some participants believe this is because of public health’s marginal position in the larger healthcare system, or because of the lack of a public health voice at the executive table. The minimal emphasis on public health deliverables in the General Letter of Expectation from the Ministry supports this contention.

Implementation is enhanced by early and extensive involvement of staff at all levels supported by formal facilitation efforts [8]. These might include, for example, high-quality training materials and on-the-job training [34]. Our data illustrate that there was almost no involvement of front line staff in the CF development process and limited manager involvement. No one mentioned training for implementing HL and STIP programs; perhaps because ‘they were already doing that’, they did not feel a need for training.

There is limited evidence to support specific KT interventions, but we know that change is more likely when KT and implementation interventions are planned and targeted to specific programs and audiences and are implemented systematically and consistently across the organization [14,38]. Although some HAs may have used KT strategies to support core program implementation, we could not identify these for HL and STIP. If KT interventions were developed, participants were not aware of them, they were not widespread across the organizations, nor did they appear to be planned and implemented in a systematic and comprehensive way.

### Summary and conclusions

The CF Framework was implemented in a complex, dynamic environment that presented significant challenges. It seems that insufficient accountability and inadequate leadership beyond public health prevented many of those responsible for implementation from doing that work and made it difficult to make it a priority. Without adequate resources, the CF Framework was perceived as a low priority, and in a chaotic environment with many competing priorities and decreasing budgets, the CF Framework became marginalized. The lack of consequences for the HAs in not implementing the programs reinforced this. Because there was no senior public health decision maker represented at the executive table in some HAs, it was difficult to promote public health renewal and secure adequate resources when higher priority was accorded to acute care and other institutionally-based health services. There were no implementation plans developed at the health authority level for such a major intervention, resulting in poor communication of the vision and intent of the Framework among staff at or near the front lines. There appeared to be no opportunities for staff and managers to consult on, or participate in developing a shared vision for the CF Framework, thus they did not feel particularly invested in the process.

The lesson to be learned is that implementation is the Achilles heel of innovation. While the Ministry of Health invested significant resources in conducting evidence reviews for all the core public health programs, they did not synthesize the evidence on factors influencing effective implementation of a complex policy intervention like the CF Framework. Unfortunately, available implementation frameworks [8,14,16-18] are rarely used prospectively to plan for implementation [16,18]. Rather, they are used primarily as we have used Greenhalgh *et al.*’s framework—retrospectively to analyze the influences on implementation.

Our main recommendation is that the implementation of any intervention requires a well developed implementation plan, guided by a comprehensive implementation framework to identify and address potential barriers and facilitators for the process. This includes involvement of staff that will be responsible for implementation in the process. Evaluation is essential to determine whether the prospective use of an implementation framework will influence successful implementation. There must be an investment of sufficient resources, not just for the activities of the intervention, but also to support the implementation process. The return on this investment is likely to be substantial.

### Consent

Written informed consent was obtained from the participant for publication of this report and any accompanying images.

## Competing interests

The authors declare that they have no competing interests.

## Authors’ contributions

LTB, RSS, and DEA took the lead in data analysis, conceptualized the thematic findings, wrote the initial draft of the paper, and approved the final version of the paper. MM, BP, and TH are the principal investigators who conceptualized the REPHS program of research and led the writing of the grant proposal for funding, from which the sections of this paper on the policy intervention and the methodology for the larger REPHS research program were derived. MM and BP participated in the data analysis, contributed to writing and editing sections of the paper, particularly the discussion, and approved the final version. TH led development of the CF Framework, contributed to the discussion section, and to editing and approving the final version of the paper. All authors read and approved the final manuscript.

## Supplementary Material

Additional file 1: Public health reform in British Columbia – framework for core functions in public health. Click here for file
