# Supplementary material for: Fading vision: knowledge translation in the implementation of a public health policy intervention
Source: Implement Sci. 2013 Jun 4;8:59. doi: 10.1186/1748-5908-8-59 (PMC3680003; doi:10.1186/1748-5908-8-59)
Supplement: Additional file 1: — Public health reform in British Columbia – framework for core functions in public health. [file 1748-5908-8-59-S1.doc]

**Public Health Reform in British Columbia –**

**Framework for Core Functions in Public Health**

This Additional File provides more detail on the larger research context for the study reported in the main article (FadingVision: Knowledge Translation in the Implementation of a Public Health Policy Intervention). To situate the study reported in the main article, we first present background on the Core Public Health Functions Framework, which is the policy intervention reported in the Fading Vision paper. We then describe the Core Public Health Functions Research Initiative (CPHFRI), a large program of public health services and systems research in British Columbia (BC), Canada.

**The Core Public Health Functions Framework**

In BC, the centrepiece of the approach to reforming and renewing the public health system is a Framework for Core Functions in Public Health [1], developed through a collaborative process involving government, health authority representatives, and other public health stakeholders. The Core Public Health Functions Framework (CF Framework) (see Figure 1) identifies the basic public health services and supports that health authorities (HAs) are expected to provide to their communities. These include: (1) twenty-one *Core Public Health Programs* in four broad areas (health improvement; disease, injury and disability prevention; environmental health; and health emergency management); (2) four *Public Health Strategies* (health promotion, health protection, preventive interventions, and health assessment/disease surveillance) to be used to implement core programs and work with communities; (3) two *Lenses*—a population lens and an equity lens applied to all core programs to ensure that health needs of particular population groups are met and to reduce health inequities; and finally, (4) *System Capacity Requirements*—those infrastructure elements necessary to support and deliver core programs, apply the population and equity lenses, and engage in public health strategies. These include, but are not limited to, competent and well-trained staff, public health information systems, public health legislation, and research to support innovation and inform policy and practice improvement [1,2]. An overview of the CF Framework and all associated evidence reviews and model core program papers can be found on the BC Ministry of Health website at: <http://www.health.gov.bc.ca/public-health/>.


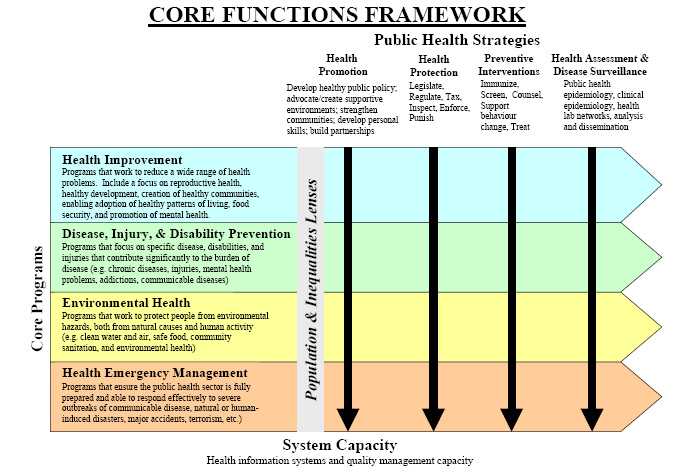


**Core Public Health Functions Research Initiative**

The launch of this Framework, which we conceptualize as a system level policy intervention, provided the impetus for developing a research program, funded by the Michael Smith Foundation for Health Research in BC and the Canadian Institutes of Health Research, to study the implementation and impact of public health system renewal, initially in BC and then in other parts of Canada. This research program, known as the *Core Public Health Functions Research Initiative* (CPHFRI: [www.uvic.ca/cphfri](http://www.uvic.ca/cphfri)), comprises several projects, including a five-year program of studies entitled *The Renewal of Public Health Systems* (RePHS), our flagship project. All CPHFRI projects study some aspect of the CF Framework implementation, and incorporate a focus on four cross-cutting themes: health equity, knowledge translation, partnerships and collaboration, and methodological innovation.

**Renewal of Public Health Systems: Additional Methodological Detail**

The larger RePHS research program, from which the data reported in the main article are derived, involves a multiple embedded case study design [3] using several sources and types of data and diverse analytic techniques. This design allows us to conduct comparative analyses capitalizing on the naturally occurring variation in public health program implementation within and between provinces. The research program encompasses five general areas of study: (1) contextual and other influences on the implementation process; (2) impacts and outcomes of implementation including the extent to which implementation is achieved; (3) the application of an equity lens to core public health programs; (4) implications of Sexually Transmitted Infection Prevention (STIP) and Healthy Living (HL) core programs for public health human resources; and (5) the nature and determinants of collaboration between the public health and primary care sectors with respect to STIP and HL.

Cases comprise the two core public health programs embedded within all six HAs from BC and six diverse health units (HU) from the province of Ontario (ON). Both the BC HAs and the ON health units reflect considerable variation in geography and location (e.g., urban vs rural), organizational structure and governance, population demographics, and economics. The data used in the analysis for this paper were obtained during phase 1 of the RePHS project, in which we studied early implementation of STIP and HL, and contextual influences on that process. We conducted interviews and focus groups with BC public health practitioners and managers involved in STIP and HL about their early implementation experiences.

Criteria for Program Selection. Because the CF Framework comprised 21 core public health programs, it was not feasible to study implementation in all of these so we selected two as exemplars, based on criteria developed with our knowledge used partners: (1) programs must have a similar focus in both provinces; (2) programs were not yet rolled out in either province at the initiation of funding so that implementation could be studied prospectively; (3) the focus of the programs were likely to involve collaboration between the public health and primary care sectors of the health system; (4) programs were not part of other CPHFRI studies. At least four core public health programs/standards met the criteria but our knowledge user partners were most interested in studying the implementation of STIP and HL.

**References**

1. British Columbia Ministry of Health Services: *A* *Framework for Core Functions in Public Health*. Victoria: 2005.
2. MacDonald M: **Developing a program of research for an Applied Public Health Chair in Public Health Education and Population Intervention Research**. *CJNR* 2011, **43**:119-124.
3. Miles MB, Huberman AM: *Qualitative Data Analysis: A Sourcebook of New Methods*. Beverly Hills, CA: Sage Publications; 1984.
